# Photothermal enhancement of chemotherapy in breast cancer by visible irradiation of Gold Nanoparticles

**DOI:** 10.1038/s41598-017-11491-8

**Published:** 2017-09-07

**Authors:** Rita Mendes, Pedro Pedrosa, João C. Lima, Alexandra R. Fernandes, Pedro V. Baptista

**Affiliations:** 1 UCIBIO, Departamento de Ciências da Vida, Faculdade de Ciências e Tecnologia, Universidade NOVA de Lisboa, Campus de Caparica, 2829-516 Caparica, Portugal; 2 LAQV/REQUIMTE, Departamento de Química, Faculdade de Ciências e Tecnologia, Universidade NOVA de Lisboa, Campus de Caparica, 2829-516 Caparica, Portugal

## Abstract

Photothermal Therapy (PTT) impact in cancer therapy has been increasing due to the enhanced photothermal capabilities of a new generation of nanoscale photothermal agents. Among these nanoscale agents, gold nanoshells and nanorods have demonstrated optimal properties for translation of near infra-red radiation into heat at the site of interest. However, smaller spherical gold nanoparticles (AuNPs) are easier to produce, less toxic and show improved photoconversion capability that may profit from the irradiation in the visible via standard surgical green lasers. Here we show the efficient light-to-heat conversion of spherical 14 nm AuNPs irradiated in the visible region (at the surface plasmons resonance peak) and its application to selectively obliterate cancer cells. Using breast cancer as model, we show a synergistic interaction between heat (photoconversion at 530 nm) and cytotoxic action by doxorubicin with clear advantages to those of the individual therapy approaches.

## Introduction

Photothermal therapy (PTT) is a minimally-invasive therapeutic strategy, where light irradiation is converted by photothermal agents to heat, thus increasing the temperature of specific tissues 1. Heating sources range from near infrared (NIR) and visible light to radiofrequency waves, microwaves, and ultrasound waves. PTT in cancer allows to selectively destroy cancer cells and spare healthy cells in the vicinity, since the former are more sensitive to an increase in temperature^1, 2^. Tissues and cells have got their natural photothermal agents, e.g. haemoglobin, cytochromes, but their absorption efficiency is very low, requiring high amounts of photon energy^1^. To enhance the photothermal effect, synthetic organic dyes are used, which may be selectively delivered to cancer cells, thus potentiating the destruction of malignant cells. Tumour environment is more hypoxic, acidic and nutrient-deficient than normal tissues, which are believed to increase the sensitivity of cancer cells to heat^3^. However, these dyes are prone to photobleaching that results in loss of anticancer activity^2^. Recently, the generation of nanoscale-based photothermal agents, such as gold nanoparticles (AuNPs), with higher absorption efficiency and without suffering from photobleaching, has relaunched PTT as an anti-cancer therapy^3^.

AuNPs exhibit unique physicochemical properties, including their surface plasmon resonance (SPR), which relies on the interaction between an electromagnetic wave and free conduction electrons at the AuNPs’ surface, causing them to oscillate coherently in resonance with the frequency of visible light, resulting in strong electromagnetic fields. This phenomenon greatly enhances both the scattering and the absorption of light by the AuNP suitable for different biomedical applications^4–6^. AuNPs are also recognised by their PTT capacities, converting electromagnetic radiation into heat due to electron excitation and relaxation^5^, which has been used for thermal ablation of tumour cells. Indeed, AuNPs of specific sizes and shapes, including gold nanorods, nanocages and nanoshells are capable to convert NIR radiation into heat^7–11^. NIR lasers are commonly used in AuNP induced PTT due to the optical window in the near-infrared, where haemoglobin, melanin and water absorption is reduced, allowing deeper light penetration into fluids and tissues^12^. Although visible light has been applied to superficial epithelial cells or in transparent organs (e.g. the eye), aiding in surgical ablation of cells or photo cauterisation of blood vessel, visible irradiation using AuNPs has had less application to cancer therapy than the NIR. Visible light penetration into the tissue is reduced to less than a millimetre, which provides for higher precision in certain medical procedures^1^. Green lasers (495–570 nm) for instance have been used for decades in medical surgery for photocoagulation in several ocular disorders as a safe tool for tissue ablation without bleeding, such as in retinoblastoma^13, 14^ focal therapy and vocal cords surgery^15, 16^. Spherical AuNPs with diameters ranging from 10 to 30 nm are ideal photothermal agents for biomedical applications since they have been shown to be non-toxic^17, 18^ and present a characteristic localised SPR band around 520 nm, i.e. in the visible region of the spectrum, with an efficient light-to-heat conversion because absorption corresponds almost totally to extinction^1, 4, 19, 20^. As such, AuNP as photothermal agents are an efficient way to induce precise heating, leading to less damages to surrounding tissues, while destroying malignant, more thermosensitive cells^4, 5^.

Thermal therapy is strongly dependent on cancer type, and tumor adaptation to temperature (i.e. thermos tolerance) may easily impact efficacy of PTT. The use of combined therapeutic approaches, relying on the synergistic interaction between heat and cytotoxic treatments has been proposed with clear advantages^3, 21^. Several reports have demonstrated that DNA damaging agents, such as Doxorubicin (DOX), are more effective in combination with hyperthermia, since DNA repair processes are temperature-dependent^21, 22^. DOX is an anthracycline compound that primarily inhibits the topoisomerase I and II and intercalate into DNA, thus inducing programmed cell death, which rapidly targets dividing cells and slow disease progression. Nevertheless, DOX toxicity in healthy cells poses major concerns in effective treatment of patients^23^.

**Figure 1:**
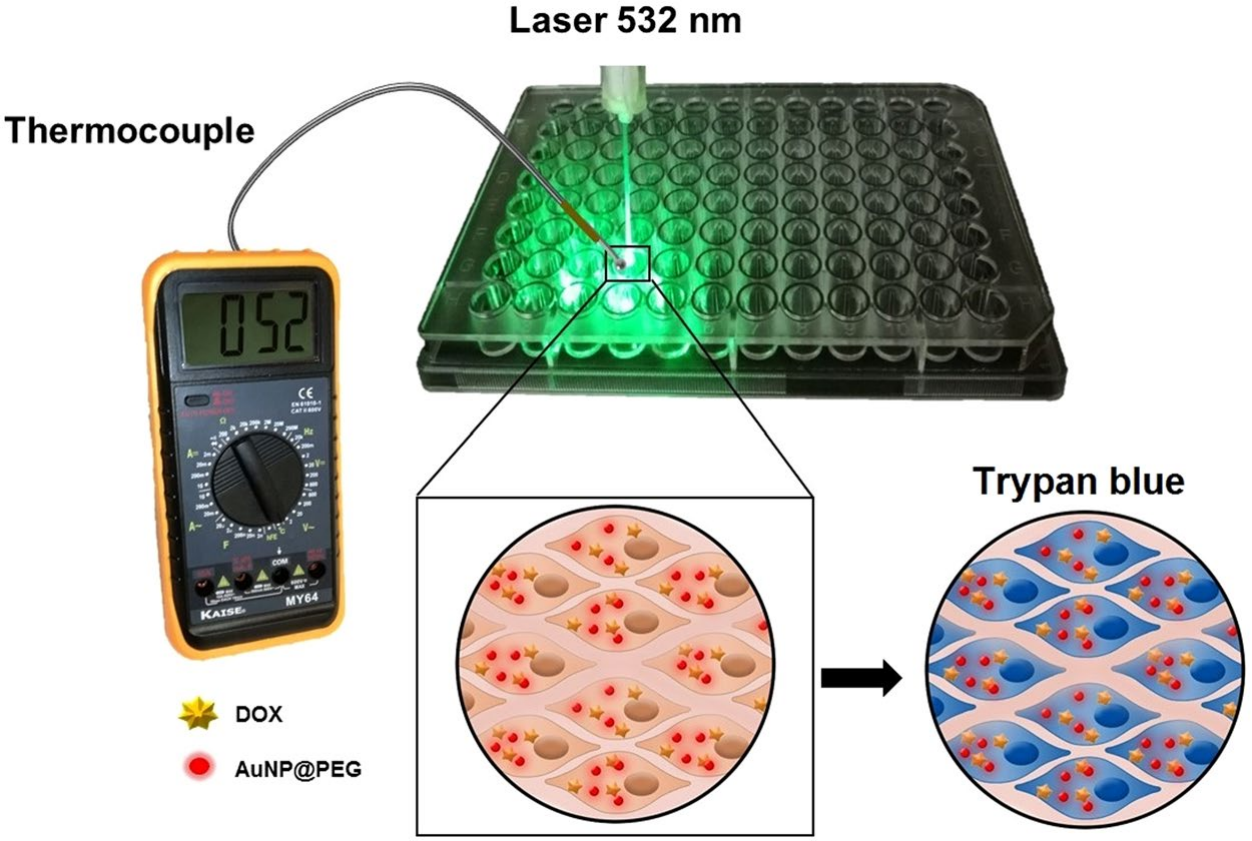
Strategy for assessing efficacy of combined therapeutics comprising doxorubicin (DOX) and AuNPs based Photothermal Therapy (PTT) in the visible. Following 6 h incubation with DOX, cells are incubated with 15 nM of AuNPs for 2 h, and then irradiated with a green laser for 60 s. Cell viability assessment via membrane integrity analysis through the trypan blue assay.

Here, we show that PTT using visible light and AuNPs as photothermal agents enhance the cytotoxic effect of DOX in breast cancer (Fig. 1). By using a traditional drug as DOX, we illustrate the possibility to easily combine chemo- and PTT with improved efficacy and doing so by irradiation with existing lasers currently used in the clinics.

## Results and Discussion

### AuNPs synthesis and functionalisation

**Figure 2:**
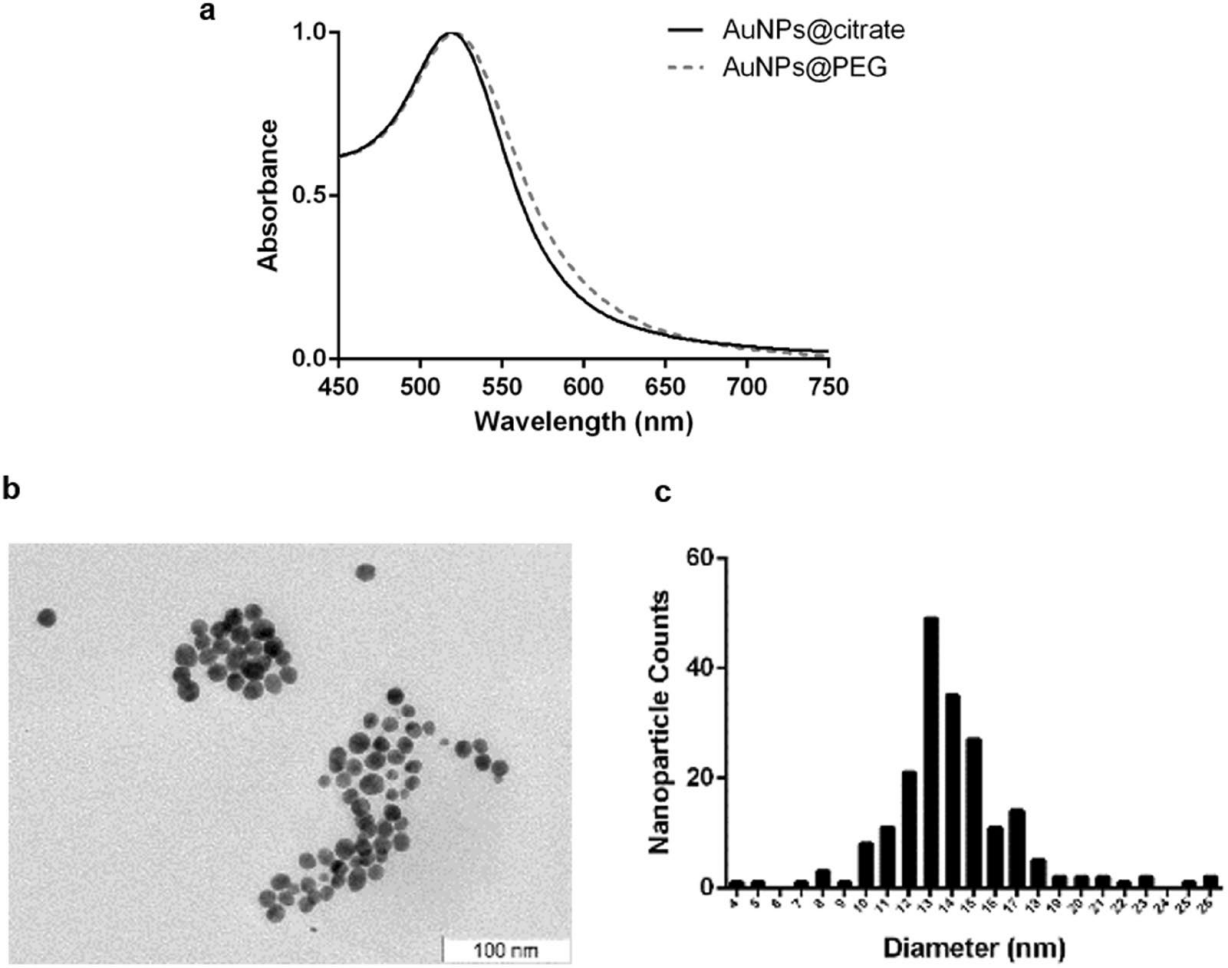
AuNPs characterisation by UV-VIS Spectroscopy and TEM. (**a**) UV-VIS spectrum of AuNPs@citrate and AuNPs@PEG, in a wavelength range of 450–750 nm, with a maximum absorption of the SPR band centred at 518 nm (AuNPs@citrate), enlightening the SPR shift from 518 nm to 521 nm. (**b**) TEM imaging (scale bar 50 nm); and (**c**) Size distribution of AuNPs@citrate (total of 200 counts) with an average diameter of 14 nm (S.D. ± 3 nm).

Stable citrate capped spherical AuNPs were synthesised with an average diameter of 14 (±3) nm, determined by TEM (Fig. 2), and further functionalised with thiolated PEG350 to saturate the AuNPs’ surface (Supplementary Fig. S1). A 100% PEG coverage renders AuNP with increased stability in biological media and improves biocompatibility. PEG350 functionalisation was confirmed by a red-shift of the plasmon absorbance peak, a 3 nm increase to the hydrodynamic radius determined by DLS and rise in Zeta Potential values of 54 mV (−74 to −20 mV) (Fig. 2 and Table 1).

**Table 1:** AuNPs characterisation by DLS and Zeta Potential Measurement.

AuNPs	Hydrodynamic Diameter (nm)	PI	Zeta Potential (mV)
AuNPs@citrate	16	0.2	−74
AuNPs@PEG	19	0.1	−20

### Laser irradiation

**Figure 3:**
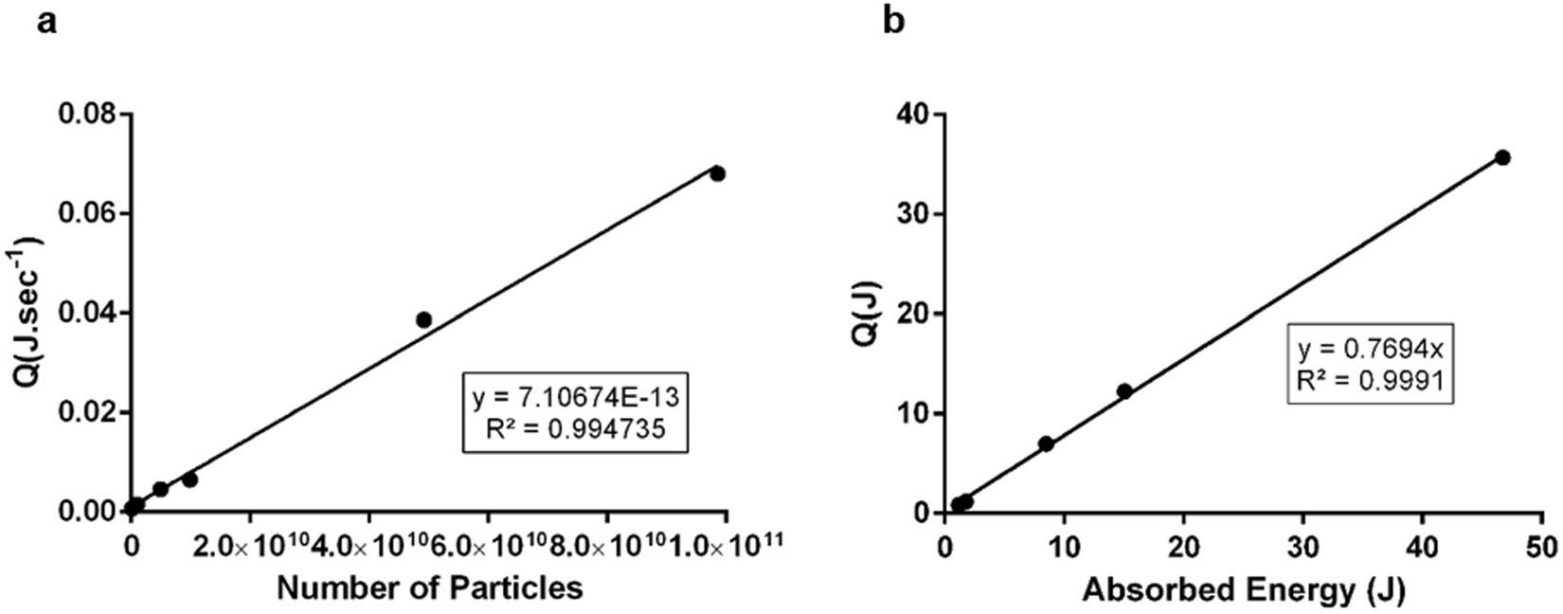
Characterisation of Photothermal Effect of Gold Nanoparticles. (**a**) Heat generated per second as function of number of particles irradiated. Several concentrations of AuNPs were irradiated at 1.7 W.cm^−2^ for 60 s and the temperature variation was measured. The slope of the curve gives the heat generated per nanoparticle per second. (**b**) Photothermal efficiency of AuNPs 14 nm.

Laser irradiation was setup as shown in Fig. 1 and the photon flux characterised by actinometry (Fig. 3). Then, the thermal effect of irradiating increasing concentrations of AuNPs@PEG (referred to as AuNPs) in water was assessed by measuring the final temperature with a thermocouple. The photothermal conversion per nanoparticle was then calculated so that the final systems could be calibrated in terms of effective hyperthermia. The rate of heat flow per particle (7.11 × 10^−13^ W) was calculated using the heat generated per assay (Q), divided by the number of AuNPs irradiated at 532 nm and total time of irradiation (see Fig. 3a). The heat was calculated, assuming a heat capacity of water of 4.18 J.g^−1^.K^−1^. The number of nanoparticles was calculated using ε = 2.85 × 10^−8^ M^−1^ cm^−1^ at 520 nm for 14 nm nanoparticles according to Navarro *et al*.^24^. The photothermal efficiency of the nanoparticles was calculated from the slope of the generated heat in function of the absorbed energy. The absorbed energy as calculated according with the equation I_A_ = I_0_ * (1–10^−A^ *)*. Dividing the absorbed laser energy by the generated heat, an efficiency of 77% photothermal conversion was determined (Fig. 3b), which is in concordance with previous studies that projected an efficiency of 78% for citrate capped 15.7 nm AuNPs^25^. This shows that 14 nm AuNPs are indeed strong visible light absorption agents capable to generate heat in a precise zone, and thus have a great potential to be applied in hyperthermia regimens^4, 5, 20, 26^.

### Photothermal Therapy induced by visible irradiation on AuNPs

AuNPs with a diameter of 14 nm are quickly taken up by cells within the first two hours of incubation, gradually slowing the uptake rate until reaching a plateau at 4–7 h^27, 28^. As such, MCF-7 cells were incubated with AuNPs (15 nM) for 2 h, and then the medium was replaced by a phenol red free DMEM medium (to avoid optical interference due to the overlap of the absorption band of phenol red) without AuNPs. MCF-7 cells were irradiated using different laser diode intensities (LDI) and increasing exposition time (see Supplementary Fig. S3 for details). The effect of hyperthermia on cell viability was evaluated by trypan blue exclusion test performed immediately after irradiation, highlighting loss of cell membranes integrity, which is severely affected by heat shock, enhancing permeability^29^. Without AuNPs and for all irradiation conditions, no trypan blue staining was observed despite an increase of the medium temperature to ~40 °C, indicating that the membranes were intact and that cells were not affected by the laser. Some reports suggest that, at this temperature, cells are thermosensitive and their membranes ought to suffer permeability changes^21^. However, in our case, no damage of cell membranes was observed for all the exposure times and laser potencies, probably due to the transient nature of the temperature increase.

**Figure 4:**
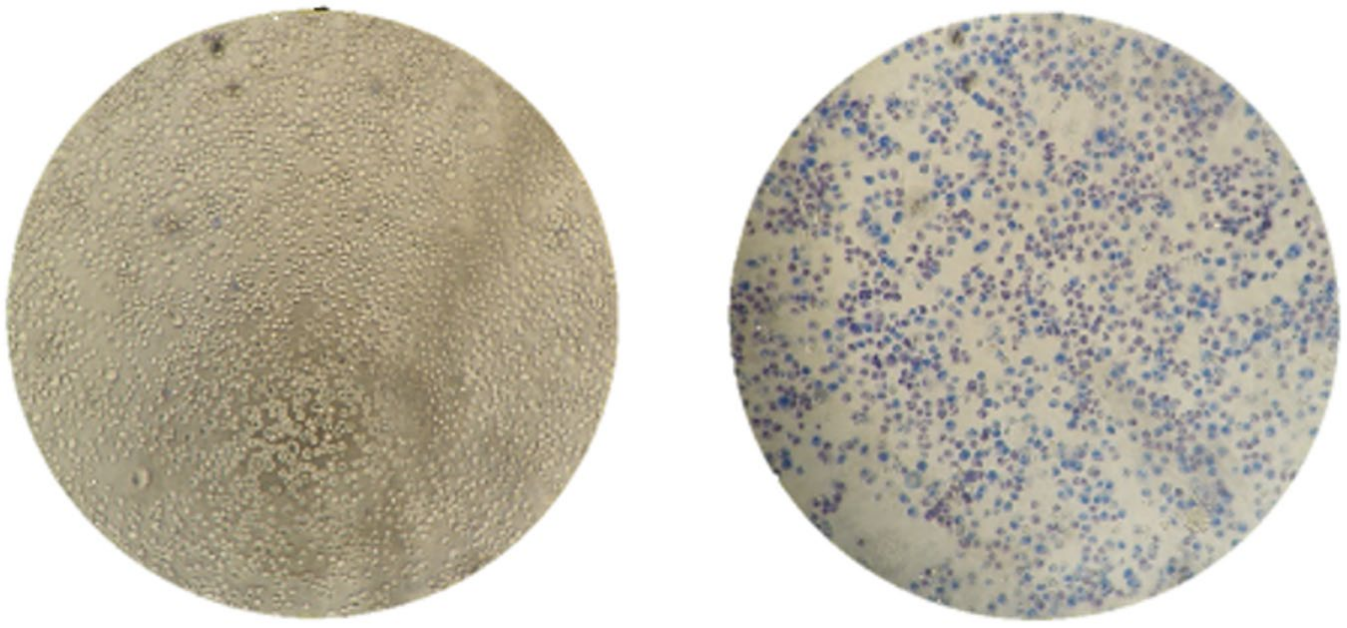
Cell fate of photothermal conversion by AuNPs in the visible. Cells irradiated in the absence (left) or presence of 15 nM of AuNPs for 2 h- PTT condition (right) - under a laser potency of 3.44 W.cm^−2^ for 60 s. In both conditions, cells were incubated with trypan Blue dye for 10 min after laser irradiation. Photos acquired using bright field (400x objective) in an inverted microscope.

Conversely, irradiation in presence of AuNPs, cells showed a strong compromise to cell membranes (Supplementary Fig. S3). Because the AuNPs are inside the cells when irradiation occurs, the intracellular surge in temperature strongly impacts cell membrane integrity, with concomitant decrease in cell viability – see Fig. 4. A laser potency of 3.44 W.cm^−2^ for 60 s was chosen for the subsequent cell assays relying on the highest temperature reached for cells irradiated with AuNPs (∆T 12 °C) compared to cells irradiated without nanoparticles.

### Combined Therapy

**Figure 5:**
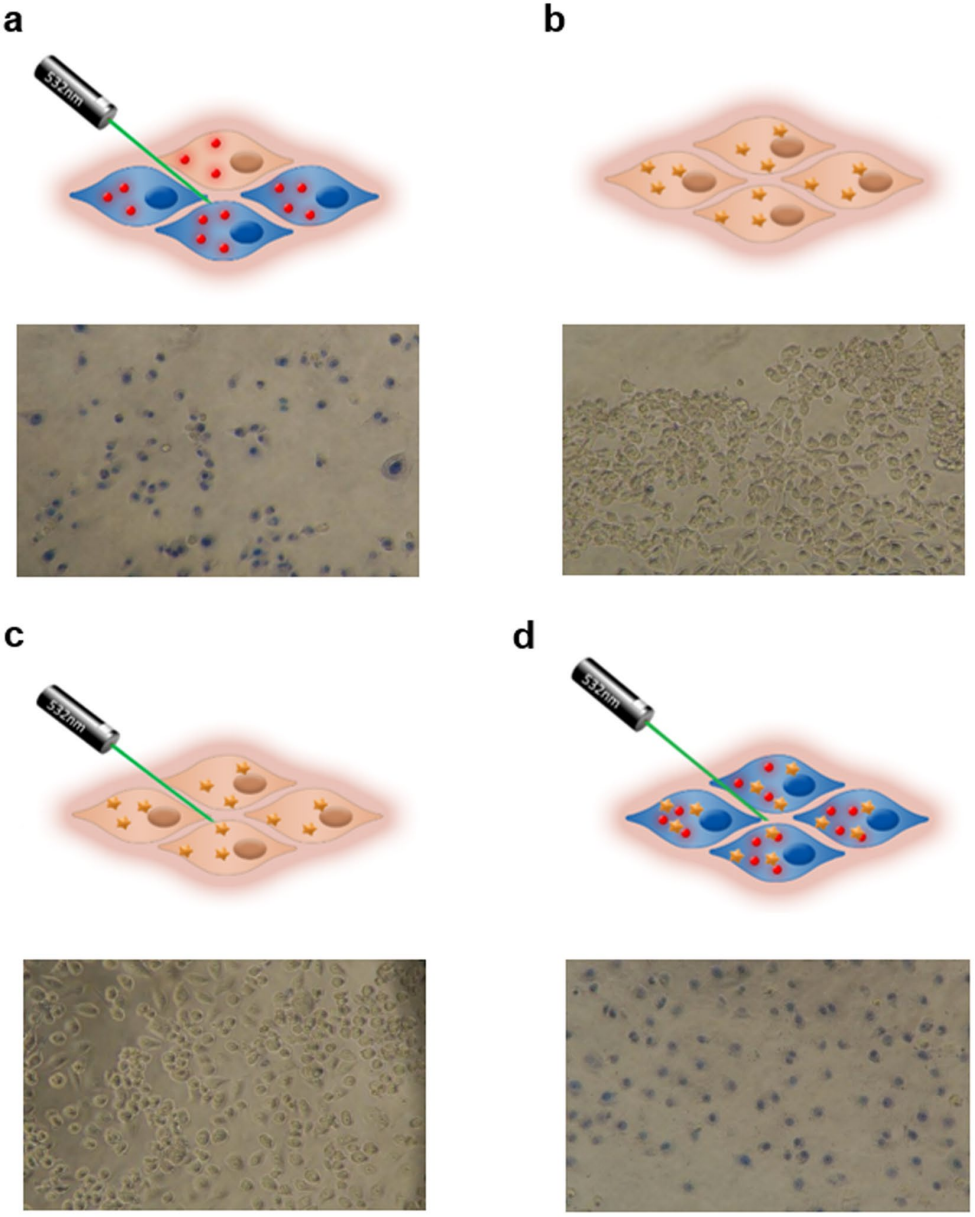
Combined chemo and PTT induced by visible light and AuNPs as photothermal agent. Schematic representation (above) and respective image after cell staining with trypan blue (800x) (below). Cells were (**a**) exposed to AuNPs (15 nM) and irradiated; challenged with DOX (3 µM) for 6 h (**b**) without or (**c**) with laser irradiation; or (**d**) combined DOX with AuNPs and irradiation. Cells were incubated with trypan blue dye for 10 min, 16 h after laser irradiation. Images acquired in bright field using an inverted microscope.

DNA repair processes, crucial for the cell’s response to aggression by cytotoxic drugs, are temperature-dependent. As such, the cell killing efficacy of antitumor drug, relying on DNA damage, see their efficacy enhanced by hyperthermia^21, 22^. DOX is one of the most widely used anticancer drugs, particularly against breast cancer^30^. As such, with the aim of improving DOX efficiency against cancer cells, we combined DOX and hyperthermia with AuNPs as heat-generator following irradiation with visible light. Cells were exposed to 3 µM DOX for 6 h (Supplementary Fig. S4), and then incubated in presence or absence of AuNPs and irradiated as depicted in Fig. 5. Membrane permeability, as indication of cell integrity and viability, was analysed using the trypan blue exclusion assay. A clear reduction in cell density was observed for cells irradiated in presence of AuNPs when compared to AuNPs alone or laser alone. This clearly indicates that the AuNPs are required for photothermal conversion of the green laser irradiation, which induces cell death followed by plate detachment (Fig. 5a). Cells challenged with DOX alone or DOX + laser irradiation showed no staining with trypan blue (Fig. 5b and c), indicating that the membrane was still intact, since there was no heat shock performed. However, when cells are challenged with DOX and AuNPs, as photothermal agents for the visible, and laser irradiation is performed, almost all cells show clear signs of losing cell integrity and extensive cell death can be observed (Fig. 5d).

**Figure 6:**
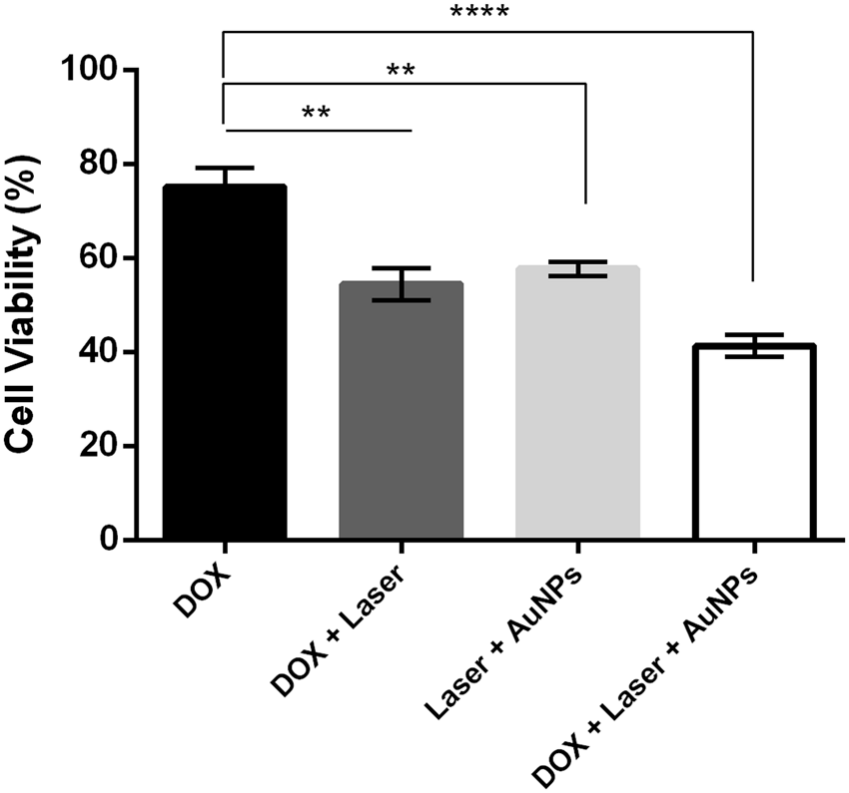
Breast cancer cell death following combined chemo and PTT induced by visible light and AuNPs as photothermal agent. Cell viability via the MTS assay in MCF-7 cells 16 h after combined therapy with DOX and AuNP induced PTT. Cells were incubated in DMEM supplemented with DOX (black bar); combination of DOX plus laser irradiation (light grey bar) (p = 0.0016); incubation with AuNPs + Laser irradiation (dark grey bar) (p = 0.0002); or combined DOX + Laser irradiation with AuNPs (white bar) (p < 0.0001). Cell viability of mono- and combined therapies normalised to the respective controls (not irradiated + 0.2% DMSO), which were set to 100%. Data are the average of quadruplicated assays and error bars correspondent to SEM (**p ≤ 0.01, ****p ≤ 0.0001).

The combined effect of AuNPs coupled to green laser irradiation and chemotherapeutic drug was further evaluated by the MTS assay in the exact same conditions as above (Fig. 5). Cells exposed to laser or AuNPs only show no decrease in viability (100%; results not shown). For DOX or photothermal alone, we observed a reduction of cell viability of 25% and 42%, respectively (Fig. 6). A synergistic reduction of 46% in cell viability with an increase of 11 °C is observed for cells challenged with the combination of DOX and AuNPs with photo irradiation. DOX combination with hyperthermia has been shown to be more effective when compared with DOX or hyperthermia alone, since DNA repair processes are temperature-dependent^21, 22^. This enhanced effect of DNA-damage has been attributed to a slowdown of the replication fork, leading to double strand breaks formation, which reduces cell survival^22^. The combined therapy - DOX with AuNPs coupled to photo irradiation –provides an additional challenge to cells, leading to 58% of viability loss with an increase in temperature of 22 °C. Because our approach allows to achieve a higher final temperature, our combined therapy showed higher reduction in MCF7 cell viability.

The apparent discrepancy between MTS vs trypan blue can be easily explained by the mechanisms of action of each therapy. Both trypan blue exclusion assay and MTS were performed 16 h after irradiation. However, only the AuNPs and laser irradiation cause membrane degradation, which remains over time. In fact, we can observe a positive trypan blue right after irradiation (Fig. 4) that remains at 16 h (Fig. 5). Our results show that only in the presence of AuNPs+ irradiation membranes seem to get compromised by the severe heat shock. This is in agreement with other studies^29^. We believe that irradiated AuNPs, especially those adsorbed to the cell membrane, promote a local fluidification and formation of pores in the membrane^31^. Regarding the MTS assay, which evaluates cell viability by correlation of the mitochondria metabolic function, we observe that DOX and DOX + laser affect mitochondria metabolism over 16 h, as expected. In fact, DOX and irradiation alone do not seem to affect membrane integrity after irradiation. Gold nanoparticles + laser results in severe local hyperthermia, which affects not only cellular membrane but also cell viability. This happens either by denaturation of proteins or nucleic acids in the cytoplasm, or by affecting cellular and\or organelles membranes^32^.

## Conclusion

Several studies have highlighted the potential use of nanomaterials for hyperthermia approach against cancer cells. Most of these reports have focused on the use of NIR to raise the temperature locally and, thus, induce cell death in a controlled way. However, despite the higher light to heat conversion of visible light upon irradiation of spherical 14–20 nm AuNPs, visible light irradiation for PTT has been neglected. In fact, only but a few reports describe the use of visible region lasers for cancer treatment, despite its extensive medical applicability^33–35^. Here, we showed that spherical AuNPs of 14 nm are perfect photothermal agents when irradiated with a green laser. In fact, a photoconversion efficiency of 77% was attained, which is much higher than conventional NIR. PTT in the visible reduced breast cancer cell viability by 60%, which was potentiated when used in combination with DOX.

As standard therapy DOX will hardly be replaced by hyperthermia alone despite the high efficiency of AuNPs+ laser (hyperthermia) in killing cancer cells. Our results support the idea that a combined therapy is more efficient against cancer cells – highest decrease in cell viability – and we believe it will be the very efficient in clinical settings. The combination of systemic and localized therapies, such as chemotherapy and hyperthermia, increases therapy efficacy not only by avoiding drug resistance but also due to the synergic effect via increased cellular uptake of drug, increased reactive oxygen species production, and increased DNA damage and inhibition of repair. By using localized hyperthermia as adjuvant therapy, we achieved a reduction in cell viability that would need higher concentrations of DOX alone for equivalent effect. However, by keeping to the same DOX concentration, we are reducing the side effects and increasing the therapeutic window.

One should point out that this is the first demonstration of the photothermal enhancement of chemotherapy in breast cancer by visible irradiation of AuNPs. Besides yielding higher temperatures, the use of AuNPs shows other advantages that have not been explored in the present report, such as the vectorization of nucleic acids, proteins and chemotherapeutics.

Combination of therapeutic approaches against cancer has become a crucial step to improve outcome and prevent the appearance of resistance to chemotherapy with concomitant impact to treatment efficacy. The exploitation of the synergistic approach herein described – photothermal ablation and chemotherapy – may circumvent these issues, since the impact to cancer cells is more severe that would prevent recovery and triggering of compensatory mechanisms. Also, such approach is clearly feasible with other photothermal agents, including anisotropic nanoparticles, such as rods and shells. Nevertheless, this study paves the way for the use of green laser irradiation combined with AuNPs in PTT regimens with particular impact in epithelial cancers. What is more, because green lasers have already been in use for surgical purposes, their combination with standard chemotherapy, allows for targeted and selective killing of cancer cells and/or the possibility of reducing the dosage of chemotherapeutic agents, without compromising efficacy but reducing side effects.

## Materials and Methods

### Biocompatible AuNP Synthesis

AuNPs with 14 (S.D. ± 3) nm were synthesised by the citrate-reduction method described by Lee and Meisel^18, 36^ and characterised by UV–VIS spectroscopy, Transmission electron microscopy (TEM) and dynamic light scattering (DLS). AuNPs functionalisation with polyethylene glycol (PEG) - AuNPs@PEG - was performed by incubating AuNP (10 nM) with 0.028% (w/v) Sodium dodecyl sulphate (SDS), and a commercial hetero-functional PEG ([O-(2-Mercaptoethyl)-O’-methyl-hexa(ethyleneglycol)], C_15_H_32_O_7_S, 356.48 Da (Sigma-Aldrich, USA)) for a period of 16 h under agitation at room temperature^37^. The excess of PEG chains was removed by centrifugation at 14000 g for 30 min at 4 °C and determination of surface saturation evaluated via Ellman’s Assay.

### Cell Culture

MCF-7 breast cancer cell line was obtained from American Type Culture Collection (ATCC, USA). Cells were maintained in DMEM medium (Dulbecco’s Modified Eagle Medium, LifeTechnologies) supplemented with 10% (v/v) Fetal Bovine Serum (FBS), 1% (v/v) of Penicillin (100 U/mL) -Streptomycin (100 μg/mL) (LifeTechnologies) and 1% MEM non-essential amino acids (Invitrogen Corp.) under an atmosphere of 5% CO_2_ and 99% relative humidity at 37 °C^38^. Upon growth to confluency, cells were trypsinised, stained with 0.4% trypan blue (Invitrogen), counted using a hemocytometer and cultured into fresh medium. Briefly, cells were seeded at a density of 2 × 10^4^ cells/well on a 96-well plate containing DMEM supplemented medium at 37 °C, 5% (v/v) of CO_2_ and an atmosphere of 99% (v/v) humidity for 24 h.

### Laser Irradiation

#### Actinometry

Prior to irradiation of cells, actinometry was performed to determine the exact amount of energy being irradiated into the system. Actinometrical measurements were performed with Aberchrome 540^TM^, E-form ((E)-a-(2,5-dimethyl-3-furylethylidene)(isopropylidene) succinic anhydride)) (Extrasynthese, France) actinometer following literature recommendations^39^. This photochromic dye is often used for actinometry studies in the near-UV and visible regions due to its reversible photocyclisation into the deep red cyclised valence isomer 7,7a-dihydro-2,4,7,7,7a-pentamethylbenzo(b)furan-5,6-dicarboxylic anhydride (C-form). When irradiated with UV light E-form turns in C-form, which can in turn be reverted to E-form when irradiated with visible light. For these measurements, a solution of 100 µM of Aberchrome 540 was dissolved in absolute ethanol and irradiated at 342 nm during 1 h until a photo-stationary state corresponding to the maximum conversion into the C-form was obtained. The C-form solution was irradiated using a continuous wave (CW) 532 nm green diode-pumped solid-state laser (DPSS) (Changchun New Industries Optoelectronics Tech. Co., LTD, Changchun, China) coupled to an optical fibre. The solution exposed to the green laser undergoes back conversion to the E-form and the number of converted molecules was quantified by UV-Vis spectroscopy (Shimadzu). For the calculation of the photon flux, a photochemical quantum yield of ϕ = 0.060 was considered (Supplementary Fig. S2) for the back reaction of C-form to E-form in ethanol.

#### Cell culture irradiation

MCF-7 cells were seeded as described above and then challenged with 15 nM AuNPs@PEG (ε = 2.85 × 10^−8^ M^−1^ cm^−1^)^24^ for 2 h. Subsequently, the culture medium was replaced by supplemented DMEM medium without phenol red pH indicator, and irradiated as described above (actinometry) – see Fig. 1. For temperature measurements, a thermocouple (Digital Multimeter, MY-64, MASTECH, Hong Kong) was inserted into the wells (in contact with the cell culture medium) before and immediately after visible light irradiation. Cell viability was immediately evaluated by trypan blue assay.

For combined therapy, MCF-7 cells were seeded and incubated at 37 °C in the absence or presence of DOX (3 µM) for 6 h, after which they were challenged with 15 nM AuNPs@PEG (ε = 2.85 × 10^8^)^24^ and irradiated (3.44 W.cm^−2^ for 60 s as described above). As controls, cells without DOX, without AuNPs@PEG and/or irradiation were assessed. Cell viability was evaluated by MTS and trypan blue assays (below) after 16 h.

### Cell Viability

*MTS assay* - Cell viability was evaluated with CellTiter 96® AQueous Non-Radioactive Cell Proliferation Assay (Promega, Madison, WI, USA), using 3-(4,5-dimethylthiazol-2-yl)-5-(3-carboxymethoxyphenyl)-2-(4-sulfophenyl)-2H-tetrazolium, inner salt (MTS) as previously described^38^. In brief, after 16 h of combined and individual therapies, cell medium was substituted by fresh medium with MTS and incubated for an additional 1h30. The absorbance was measured at 490 nm (the absorbance of a well without cells was subtracted to all conditions) and the following formula applied to calculate the cell survival rate (eq. 1):

1 $$Cell\,Viability\,( \% )=\frac{({\rm{mean}}\,{\rm{Abs}}.\,{\rm{of}}\,{\rm{treatment}}\,{\rm{group}})}{({\rm{mean}}\,{\rm{Abs}}.\,{\rm{of}}\,{\rm{control}}\,{\rm{group}})}\times 100$$

*Trypan blue exclusion assay* – Cells were incubated with 100 µL of trypan blue 0.4% (m/v) solution (Invitrogen) for 10 minutes, washed three times with DMEM without phenol red, imaged in bright field inverted microscope (Nikon TMS, Tokyo, Japan) and pictures were taken using a Digital Camera (Sony RX100 MK2, Japan).

### Statistics

Statistical significance of all data was verified by One-way ANOVA. The Tukey method allowed to determine statistically significant differences between mono and combined therapeutics. This analysis was performed with GraphPad Prism 6.0 (GraphPad Software, Inc) and results were considered statistically significant for p < 0.05. Data are the average of quadruplicated assays and the errors are calculated by the standard error mean.

## Electronic supplementary material

Supplementary Information

## References

[1] Cabral RM, Baptista PV (2013). The Chemistry and Biology of Gold Nanoparticle-Mediated Photothermal Therapy: Promises and Challenges. Nano Life.

[2] Huang X, El-Sayed MA (2011). Plasmonic photo-thermal therapy (PPTT). Alexandria. J. Med..

[3] Abadeer NS, Murphy CJ (2016). Recent Progress in Cancer Thermal Therapy using Gold Nanoparticles. J. Phys. Chem. C.

[4] Cabral RM, Baptista PV (2014). Anti-cancer precision theranostics: a focus on multifunctional gold nanoparticles. Expert Rev. Mol. Diagn..

[5] Huang X, El-Sayed MA (2010). Gold nanoparticles: Optical properties and implementations in cancer diagnosis and photothermal therapy. J. Adv. Res..

[6] Sharma H, Mishra PK, Talegaonkar S, Vaidya B (2015). Metal nanoparticles: a theranostic nanotool against cancer. Drug Discov. Today.

[7] Hirsch LR (2003). Nanoshell-mediated near-infrared thermal therapy of tumors under magnetic resonance guidance. PNAS.

[8] Elbialy N, Abdelhamid M, Youssef T (2010). Low Power Argon Laser-Induced Thermal Therapy for Subcutaneous Ehrlich Carcinoma in Mice Using Spherical Gold Nanoparticles. J. Biomed. Nanotechnol..

[9] El-Sayed IH, Huang X, El-Sayed MA (2006). Selective laser photo-thermal therapy of epithelial carcinoma using anti-EGFR antibody conjugated gold nanoparticles. Cancer Lett..

[10] Huang X, Jain PK, El-Sayed IH, El-Sayed M (2006). a. Determination of the Minimum Temperature Required for Selective Photothermal Destruction of Cancer Cells with the Use of Immunotargeted Gold Nanoparticles. Photochem. Photobiol..

[11] Huang X, Qian W, El-Sayed IH (2007). & El-Sayed, M. a. The potential use of the enhanced nonlinear properties of gold nanospheres in photothermal cancer therapy. Lasers Surg. Med..

[12] König K (2000). Multiphoton microscopy in life sciences. J. Microsc..

[13] Yan Y, Olszewski AE, Hoffman MR, Zhuang P (2010). Use of Lasers in Laryngeal Surgery. J Voice.

[14] Zeitels SM (2011). Local Injection of Bevacizumab (Avastin) and Angiolytic KTP Laser Treatment of Recurrent Respiratory Papillomatosis of the Vocal Folds: A Prospective Study. Ann. Otol. Rhinol. Laryngol..

[15] Kozak I, Luttrull JK (2015). Modern retinal laser therapy. Saudi J. Ophthalmol..

[16] Cheng, J. & Buys, Y. M. *Lasers in Open Angle Glaucoma*. 50–53, doi:10.17925/USOR.2014.07.01.50 (2014).

[17] Conde J (2014). Gold-nanobeacons for gene therapy: evaluation of genotoxicity, cell toxicity and proteome profiling analysis. Nanotoxicology.

[18] Conde, J., Rosa, J. & Baptista, P. Gold-Nanobeacons as a theranostic system for the detection and inhibition of specific genes. Protoc. Exch. 1–35, doi:10.1038/protex.2013.088 (2013).

[19] Jain S, Hirst DG, O’Sullivan JM (2012). Gold nanoparticles as novel agents for cancer therapy. Br. J. Radiol..

[20] Qin Z (2016). Quantitative Comparison of Photothermal Heat Generation between Gold Nanospheres and Nanorods. Sci. Rep..

[21] Wust P (2002). Hyperthermia in combined treatment of cancer. The Lancet-Oncology.

[22] Schaaf L (2016). Hyperthermia synergizes with chemotherapy by inhibiting PARP1-dependent DNA replication arrest. Cancer Res..

[23] Tacar O, Sriamornsak P, Dass CR (2013). Doxorubicin: An update on anticancer molecular action, toxicity and novel drug delivery systems. J. Pharm. Pharmacol..

[24] Navarro JRG, Werts MHV (2013). Resonant light scattering spectroscopy of gold, silver and gold–silver alloy nanoparticles and optical detection in microfluidic channels. Analyst.

[25] Jiang K, Smith DA, Pinchuk A (2013). Size-Dependent Photothermal Conversion Efficiencies of Plasmonically Heated Gold Nanoparticles. J. Phys. Chem. C.

[26] Raji V (2011). Selective photothermal efficiency of citrate capped gold nanoparticles for destruction of cancer cells. Exp. Cell Res..

[27] Chithrani BD, Ghazani AA, Chan WCW (2006). Determining the size and shape dependence of gold nanoparticle uptake into mammalian cells. Nano Lett..

[28] Moser F (2016). Cellular Uptake of Gold Nanoparticles and Their Behavior as Labels for Localization Microscopy. Biophys. J..

[29] Richter K, Haslbeck M, Buchner J (2010). Review The Heat Shock Response: Life on the Verge of Death. Mol. Cell.

[30] American Cancer Society - Chemotherapy for Breast Cancer. at https://www.cancer.org/cancer/breast-cancer/treatment/chemotherapy-for-breast-cancer.html.

[31] St-Louis Lalonde B (2013). Visible and near infrared resonance plasmonic enhanced nanosecond laser optoporation of cancer cells. Biomed. Opt. Express..

[32] Hildebrandt B (2002). The cellular and molecular basis of hyperthermia. Crit. Rev. Oncol. Hematol..

[33] Dierickx CC, Anderson RR (2005). Visible light treatment of photoaging. Dermatol. Ther..

[34] Azadgoli B, Baker RY (2016). Laser applications in surgery. Ann. Transl. Med..

[35] Schulze M (2010). Medical Applications of Lasers: Diversity is Key to Success. Laser iTechnik J..

[36] Lee, C. & Meisel, D. Adsorption and Surface-Enhanced Raman of Dyes on Silver and Gold Sols’. 60439, 3391–3395 (1982).

[37] Conde J, Rosa J, de la Fuente JM, Baptista PV (2013). Gold-nanobeacons for simultaneous gene specific silencing and intracellular tracking of the silencing events. Biomaterials.

[38] Fernandes AR (2017). Multifunctional gold-nanoparticles: A nanovectorization tool for the targeted delivery of novel chemotherapeutic agents. J. Control. Release.

[39] Rappon M, Syvitski RT (1996). Kinetics of photobleaching of Aberchrome 540 in various solvents: solvent effects. J. Photochem. Photobiol. A Chem..

