# Supplementary material for: Photothermal enhancement of chemotherapy in breast cancer by visible irradiation of Gold Nanoparticles
Source: Sci Rep. 2017 Sep 7;7:10872. doi: 10.1038/s41598-017-11491-8 (PMC5589803; doi:10.1038/s41598-017-11491-8)
Supplement: Supplementary file 1 — Supplementary Information [file 41598_2017_11491_MOESM1_ESM.pdf]

Photothermal enhancement of chemotherapy in breast cancer by visible irradiation of Gold Nanoparticles

Rita Mendes[1], Pedro Pedrosa[1], João C. Lima[2], Alexandra R. Fernandes[1,*], Pedro V. Baptista[1,*]

[1]UCIBIO, Departamento de Ciências da Vida, Faculdade de Ciências e Tecnologia, Universidade NOVA de Lisboa, Campus de Caparica, 2829-516 Caparica, Portugal

[2]LAQV/REQUIMTE, Departamento de Química, Faculdade de Ciências e Tecnologia, Universidade NOVA de Lisboa, Campus de Caparica, 2829-516 Caparica, Portugal

*Corresponding authors: Alexandra R. Fernandes (ma.fernandes@fct.unl.pt) and Pedro V. Baptista (pmvb@fct.unl.pt), UCIBIO, Departamento de Ciências da Vida, Faculdade de Ciências e Tecnologia, Universidade NOVA de Lisboa, 2829-516 Caparica, Portugal. Tel/Fax: +351-21-2948530.

Supporting Data

AuNPs Characterization

A **B**

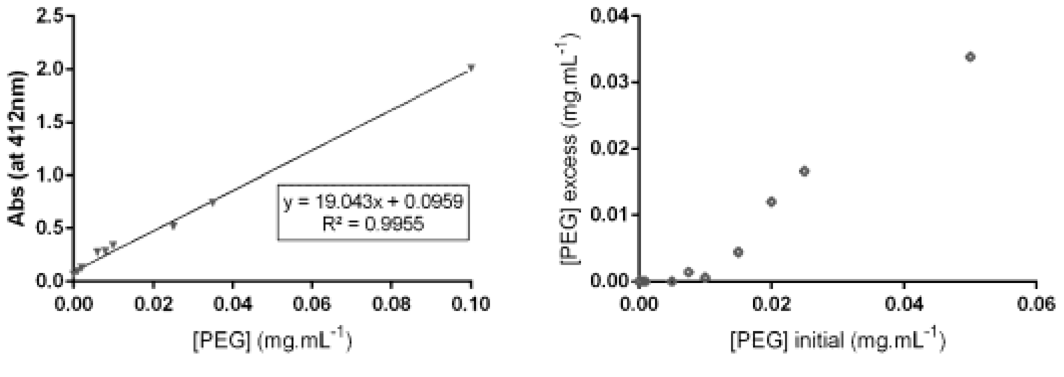

Figure S1 - Determination of the degree of saturation of AuNPs functionalization with PEG. (A) Standard calibration curve of PEG chains obtained by UV-visible spectroscopy. PEG concentrations were plotted with the absorbance at 412 nm and the resulting equation

allows the determination of unknown PEG concentrations. **(B) Variation of the excess of PEG chains as function of initial concentration in the incubation with a fixed concentration of AuNPs (10 nM)**. At 0.01 mg.mL-1 of initial PEG concentration the 100 % degree of saturation is achieved and for higher concentrations, PEG chains are detected in the supernatants.

Photothermal efficiency

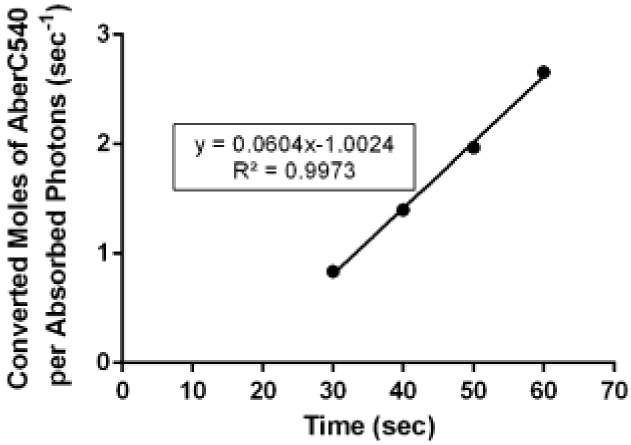

Figure S2 - A) Quantum yield of Aberchrome 540^{TM} in Ethanol. A solution of 100 µM of Aberchrome 540 was dissolved in absolute ethanol and irradiated at 342 nm during 1 h until a photo-stationary state corresponding to the maximum conversion into the C-form was obtained. The C-form solution was then exposed to a light source of 532 nm for intervals of 10 seconds and the absorption spectrum was calculated. The number of molecules of the converted actinometer were divided by the number of photons absorbed and plotted over time. A quantum yield of 0.06 % was obtained from the slope of the curve for the back reaction of C-form to E-form in ethanol.

Optimization of AuNPs-induced Photothermal Therapy in cells

LDI / Exposure Time	Cells only	Cells + AuNPs (PTT condition)
3.99W.cm^{-2} / 30s (total=33.7J)	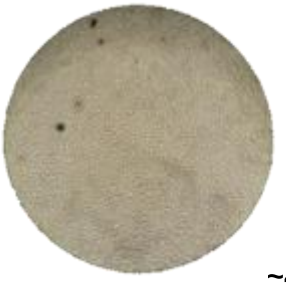 ~42 ºC	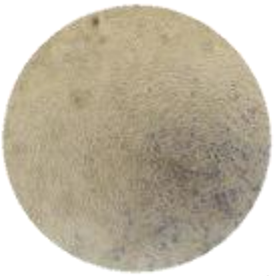 ~50 ºC
2.37 W.cm^{-2} 60s (total = 47J)	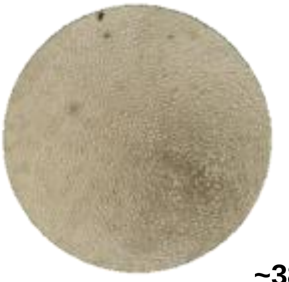 ~38 ºC	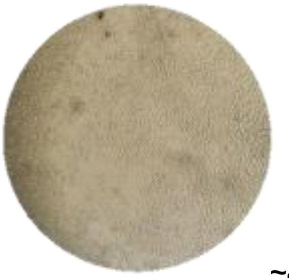 ~43 ºC
3.44W.cm^{-2} / 60s (total = 51.1J)	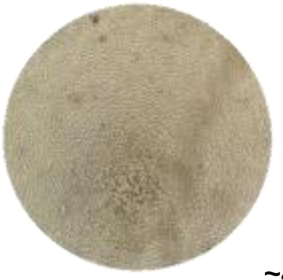 ~40 ºC	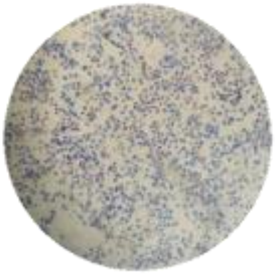 ~52 ºC
3.99W.cm^{-2} / 60s (total=67.3J)	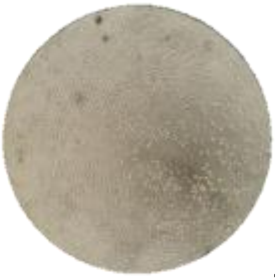 ~48 ºC	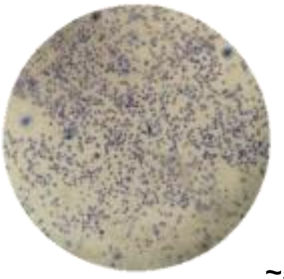 ~58 ºC

**2.9W.cm^{-2} / 90s (total = 67.6J)**	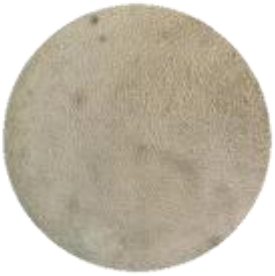 **~43 ºC**	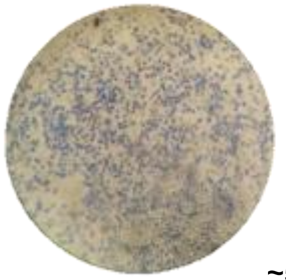 **~52 ºC**
**3.44W.cm^{-2} / 90s (total = 67.6J)**	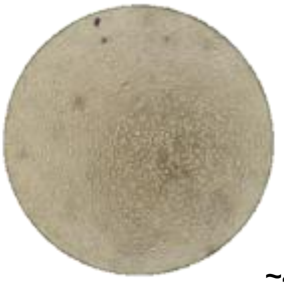 **~48 ºC**	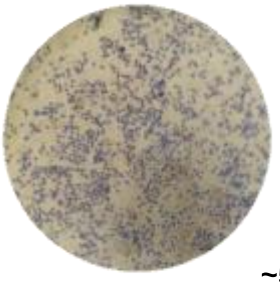 **~56 ºC**
**2.37W.cm^{-2} / 120s (total = 93.8J)**	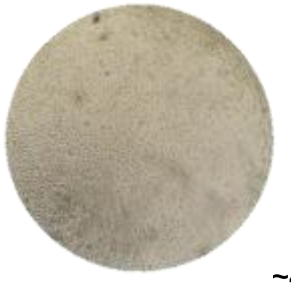 **~43 ºC**	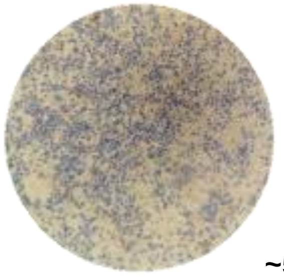 **~55 ºC**
**2.9W.cm^{-2} / 120s (total = 114.8J)**	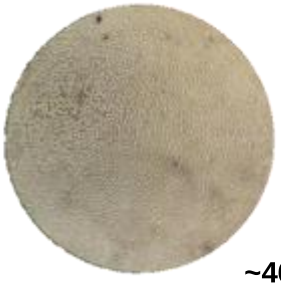 **~46 ºC**	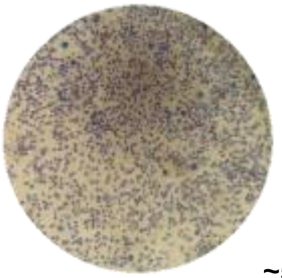 **~58 ºC**

Figure S3 - Images of Irradiated Cells (only) and Cells + AuNPs (PTT condition) under different LDIs and exposure times. Cells were incubated with 15 nM of AuNPs for 2 h or DMEM and irradiated. In all conditions cells were incubated with Trypan Blue dye for 10 min and pictures were taken right after in bright field (400x objective) using an inverted microscope (final achieved temperatures are indicated at the bottom right in each picture).

Chemotherapy

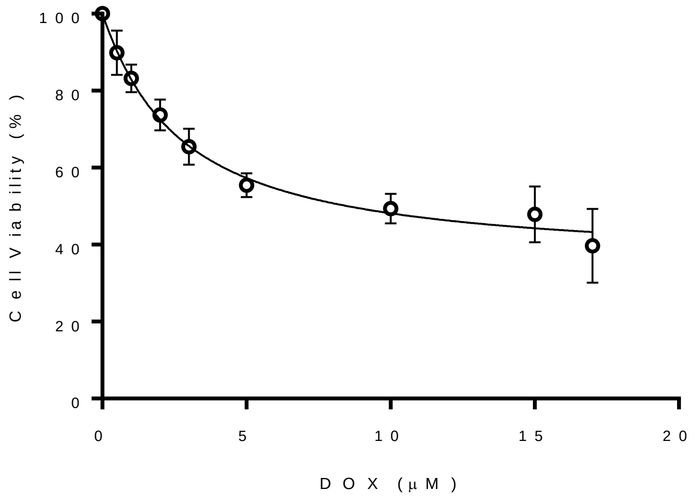

Figure S4 - Dose dependent cytotoxicity of DOX in MCF-7 cells. Cell viability values were normalized to the control (cells incubated with 0.2 % DMSO), which were set to 100 %. Data are the average of trip assays and error bars correspondent to SEM.

According to previous reports, IC_{50} = 3 µM is within the calculated intervals for MCF-7 cell line (1-5 µM) [36]. Despite being determined by two different techniques (MTS and MTT), the conditions of the assay were the same (DOX has been exposed to cells for 48 h in both cases).

DOX uptake by cells is very quick, which allows a half-life distribution in 3-5 min. Despite that, DOX takes longer to be eliminated from the tissue, having a terminal half-life of 24-36 h [1]. Considering its fast distribution, an incubation period of 6 h was tested with the estimated relative IC_{50} (48 h).
